# The Use of Stem Cells in Bone Regeneration of Cleft Lip and Palate Patients: A Systematic Review

**DOI:** 10.3390/jcm13175315

**Published:** 2024-09-08

**Authors:** Mohamed Jaber, Aalaa Majed Alshikh Ali, Roba Imad El Saleh, Prathibha Prasad

**Affiliations:** 1 Department of Clinical Dental Sciences, College of Dentistry, Ajman University, Ajman P.O. Box 346, United Arab Emirates; mohamed.jaber@ajman.ac.ae; 2 Center of Medical and Bio-Allied Health Sciences Research, Ajman University, Ajman P.O. Box 346, United Arab Emirates; 3 Interns, College of Dentistry, Ajman University, Ajman P.O. Box 346, United Arab Emirates; 201910880@ajmanuni.ac.ae (A.M.A.A.); 201910512@ajmanuni.ac.ae (R.I.E.S.); 4 Department of Basic Medical and Dental Sciences, College of Dentistry, Ajman University, Ajman P.O. Box 346, United Arab Emirates; 5 Department of Oral Pathology, Saveetha Dental College and Hospital, Saveetha Institute of Medical and Technical Sciences (SIMATS), Saveetha University, Chennai 600077, India

**Keywords:** cleft palate, cleft lip, alveolar cleft, stem cell, graft, regenerative, reconstruction

## Abstract

**Background and Objectives:** Cleft lip alone or a combination of cleft lip and palate (CLP) is a common developmental abnormality in the craniofacial region. This umbrella review aims to identify promising avenues for treatment using stem cell therapy. **Materials and Methods:** Systematic reviews from 2014 to 2024 were searched among databases like PubMed, Medline, and Google Scholar. PRISMA guidelines were employed to ensure the thoroughness of the search. A quality assessment (ROBIS) of the included reviews was conducted to ensure the reliability and validity of the synthesized evidence. **Results:** Five systematic reviews were selected for this umbrella review. Results show that stem cell therapy, specifically using mesenchymal stem cells (MSCs) and adipocyte stem cells (ADSCs), promotes bone regeneration in CLP deformities. Although multiple studies have established the effectiveness of diverse types of stem cells in treating CLP, important considerations including safety concerns, methodological variability, and the need for standardization have been identified. The fact that the number of relevant systematic reviews that matched our inclusion criteria was limited could affect this research’s robustness and may limit the breadth and depth of evidence synthesis. Definitive conclusions could not be reached due to variation among treatments and outcomes. **Conclusions**: The examined studies highlight the potential of stem cell therapy as a complementary approach to existing treatments for CLP. However, there are challenges that need to be addressed, including concerns regarding safety, variations in methodologies, and the need for standardization. Exploring the potential of other stem cell types may further enhance treatment outcomes for CLP patients.

## 1. Introduction

Cleft lip alone, or in occurrence with the cleft of the palate (CL/P), is the most common developmental anomaly in the craniofacial region, with an estimated incidence of around 1 in 700 infants born alive [1]. Clefts occur during the fourth stage of development. The locations at which the fusion of different developmental processes fails to occur indicate the impacted site. A cleft lip develops due to failure in the fusion of the frontonasal and maxillary processes, and failure in the fusion of the secondary palatal shelves of the maxillary process leads to a cleft palate [2]. Based on the International Perinatal Database of Typical Orofacial Clefts (IPDTOC), a total of 9.92 cases of cleft lip per 10,000 births, with or without cleft palate, was reported. A total of 3.28 per 10 thousand people had an isolated cleft lip, while 6.64 per 10 thousand people had a cleft lip and palate association [3].

Although genetic factors are a major contributor to the etiology of cleft lip and palate, external factors such as hormonal imbalance, drug use, malnutrition, exposure to toxins, and a variety of other biological factors are also involved in their etiology [4,5]. There are regional and national differences in the overall incidence of orofacial clefts. Racial and cultural diversity, differences in climatic conditions, and variations in programs for the care of expectant mothers could all contribute to this disparity [6]. These patients are prone to various complications, including psychosocial distress due to compromised esthetics, speech and hearing impairments, and feeding difficulties [7]. 

The therapeutic journey for cleft lip and/or palate patients begins as early as three months old, involving numerous maxillofacial reconstructions, with the first being lip repair. Palatal closure surgery is meticulously determined by the surgeon and is typically performed when the child is between six and twelve months old [8,9,10].

Using stem cells in cleft therapy has a promising effect on bone regeneration and is considered an adequate alternative to conventional bone grafting techniques. Clonogenic cells with the capacity to divide, multiply, and differentiate are identified as stem cells. Adult stem cells, induced pluripotent stem cells (iPSCs), and embryonic stem cells are the three distinct categories of stem cells [11]. Adult stem cells are multipotent or unipotent and are found in different tissues, including the skin, blood, intestine, muscle, heart, and brain. Their main function is maintaining tissue hemostasis, and in case of injury or loss of cells, they can proliferate and self-renew themselves [12]. Adult stem cells are divided into hematopoietic stem cells and mesenchymal stem cells [13]. By genetically modifying adult somatic stem cells to transform into embryonic stem cells, induced pluripotent stem cells (iPSCs) can be created [14]. iPSC-derived tissues closely resemble the cell donor, which is a critical factor in disease modeling and drug screening research [13]. Furthermore, non-hematopoietic mesenchymal stem cells (MSCs) possess multipotency and can differentiate into the mesodermal (osteocytes, adipocytes, and chondrocytes), ectodermal (neurocytes), and endodermal (hepatocytes) lineages [15].

MSCs are a type of stem cell that is widely studied in the field of regenerative drugs and are found in copious quantities in adipose tissue. Following liposuction or lipectomy, they can be exfoliated via aspiration [13]. Because of their diverse spectrum of differentiation potentials, adipose stem cells have been considered for application in the field of regenerative medicine, specifically in relation to bone regeneration. One of their main applications is orofacial bone regeneration [13]. 

Most embryonic stem cells originate from fertilized eggs in a vitro clinic, rather than naturally fertilized eggs in vivo clinics [16]. The groundbreaking discovery of iPSCs enabled researchers to ethically experiment on pluripotent stem cells without the need to face controversies regarding the use of embryos [17]. There are numerous disagreements when it comes to the gold standard among stem cells. Bone marrow stem cells are by far the most-often-employed stem cells to treat patients with cleft palates. Bone marrow stem cells consist of both hematopoietic and mesenchymal stem cells and are harvested from the iliac crest or sternum [18]. According to some authors, embryonic stem cells are still widely regarded as the “gold standard” [19]. However, some other authors believe that, from an ethical and moral standpoint, adult embryonic stem cells are recognized as the gold standard of care in clinical trials. According to Prentice, adult stem cells are the real gold standard in regenerative medicine [20]. Adult stem cells and MSCs are present throughout the body and were first discovered by Friedenstein in 1976. These cell source, which was originally discovered in bone marrow, is believed to be the best for clinical research according to him [21]. Another study mentioned that, based on their multipotency and capacity for renewal, bone marrow MSCs are presently the gold standard [22]. However, according to another study, a comparison is also made between adipose stem cells and bone marrow stem cells, which are still regarded as the gold standard of stem cells [23]. Mesenchymal stem cells generated from bone marrow are considered gold-standard osteoprogenitors according to studies by Le et al. However, they also argued that several factors related to bone marrow-derived mesenchymal stem cell therapies remain unclear, such as the use of pure cell preparations, the substantial number of cells needed to accomplish satisfactory healing, the need for growth factor supplementation, and the incomplete healing of fractures in many patients. Further disadvantages that discourage interest in their therapeutic application involve the invasive nature of isolating mesenchymal stem cells, their extremely low frequency in bone marrow, and the need for copious quantities to promote bone regeneration. Hence, they mentioned that, as opposed to BMMSCs, adipose-derived stem cells have the following benefits: adipose-derived stem cells have a greater proliferative capacity, can be separated in large quantities using a straightforward process, and can hold onto their differentiation potential for an extended amount of time [24]. A “golden approach” cannot be identified because of the variety of study designs, and not all studies produced satisfactory results. However, most studies mentioned adult stem cells, mesenchymal stem cells, as the recent gold standard that could be used without facing ethical controversies. The study aims to examine the role and effectiveness of stem cells in the treatment of cleft lip and palate (CLP) and highlight areas that require further investigation and improvement.

## 2. Material and Methods

### 2.1. Research Question

This umbrella review aims to assess the efficiency of employing stem cell-based interventions in promoting bone regeneration for the remediation of cleft lip and palate deformities using the following PICO questions:

- Population: Individuals with cleft lip and palate;
- Intervention: Use of stem cells for bone regeneration;
- Comparison: Other regenerative strategies available;
- Outcome: The effectiveness of utilizing stem cells for bone regeneration in repairing cleft lip and palate.

### 2.2. Search Strategy and Eligibility Criteria

**Figure 1:**
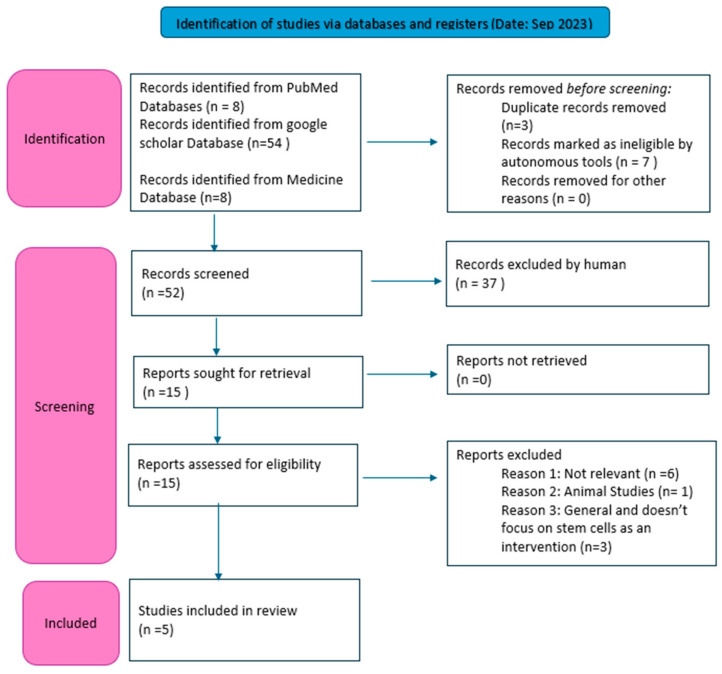
PRISMA flow diagram.

A systematic literature review/an electronic search was conducted using the electronic databases PubMed, Medline, and Google Scholar. The search keywords used were “stem cell/cells”, “cleft palate”, “graft/grafts”, “bone regeneration”, “cleft lip/lips”, “regeneration”, “regenerative”, “reconstruction”, “alveolar cleft”, and “cleft defect”. Table 1 lists the inclusion and exclusion criteria that were applied. The PRISMA flow chart demonstrated in Figure 1 demonstrates the selection process of the retrieved articles.

### 2.3. Study Selection and Data Collection

The search and study selection processes were conducted by two reviewers (A.A. and R.E.). In case of any disagreement on a study’s eligibility, it was resolved by a third reviewer (M.J.). Initial screening based on titles and abstracts was independently performed by the reviewers, followed by a subsequent assessment of full texts for potential inclusion. Any disagreements during this process were resolved with the third reviewer.

### 2.4. Risk of Bias (RoB) Assessment

**Figure 2:**
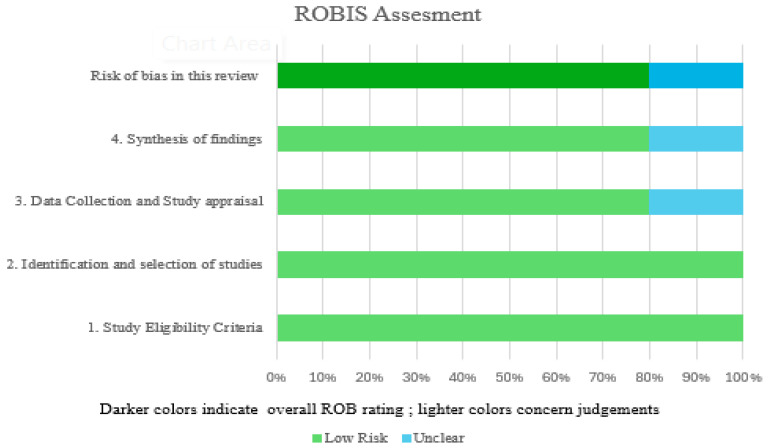
ROBIS results of included studies.

Three reviewers (P.P., A.A., and R.E.) initially screened the titles and abstracts relevant as per the inclusion criteria. The PRISMA flow chart recorded the data collection. After removing all duplicate copies, the authors retrieved the selected article with its full text. Only five articles were finally selected for this review and independently reviewed. A fourth reviewer, M.J., evaluated the studies with disagreement. All the finalized articles were included in Table 2. The risk of bias in the included studies is shown in Figure 2.

This study was registered in the PROSPERO database (CRD42024566111). This work is an umbrella review of several systematic reviews with varying techniques for treating the clefts.

## 3. Results

### 3.1. Search Results

Figure 1 portrays a flowchart illustrating the technique used in this umbrella review. A thorough search of multiple databases yielded 70 articles. After screening based on title and abstract, 15 full-text papers were evaluated for eligibility. Following the first screening, six publications were eliminated for several reasons. Two of them were irrelevant to the current study, while one focused only on animal studies. Furthermore, three studies presented interventions that did not focus solely on stem cells. These exclusions were critical in refining the selection process, ensuring that only relevant and appropriate studies were included for further analysis. Five systematic reviews underwent qualitative analysis. 

### 3.2. Characteristics of the Selected Studies

A systematic review by Badr et al. investigated adipocyte stem cells (ADSCs) used in the treatment of cleft lip and palate. Four studies were included. The study yielded important findings regardless of the limited number of articles included, suggesting promising outcomes in managing cleft patients. The evidence was not enough to warrant its routine use by oral surgeons; however, their efficiency in bone regeneration and reconstruction has been highlighted in the study. However, the study highlighted the potential of tumor formation from the human ADSCs and recommended that their safety testing be mandatory [25].

Abbasi et al., in a systematic review, investigated various uses of stem cells in dentistry and described their current state in intraoral applications, and included 172 studies, 16 of which are human studies. Insufficient human studies exist involving stem cell therapy for dental regeneration. Clinically, researchers were able to regenerate periodontal-like tissue, bone, and pulp-like tissue, but complete tissue regeneration is still difficult. The study concluded that stem cell treatment in dental applications has been notably promising. Combining in vitro osteogenic-differentiated human bone marrow mesenchymal stem cells (hBMMSCs) with recombinant platelet-derived growth factor enhanced bone regeneration, particularly during alveolar cleft repair [26].

Sepanta and colleagues’ goal was to evaluate the effectiveness of various scaffolds when combined with growth hormones and stem cells in both human and animal models. Among the 46 studies reviewed, combining scaffolds, mesenchymal stem cells (MSCs), and growth factors resulted in considerable bone repair. Notably, employing human bone marrow MSCs in conjunction with calcium sulfate resulted in bone regeneration rates of 35.4% and 25.6% after four months. Furthermore, HA/TCP loaded with MSCs and platelet-rich growth factor demonstrated a 51.3% mean bone formation after three months and a comparable bone formation (41.34%) after 12 months. Furthermore, combining PDGF with HA/TCP and MSCs improved bone growth in anterior maxillary clefts [27].

Martín et al. systematically reviewed and evaluated the effectiveness of stem cells for bone repair in individuals with alveolar bone atrophy. Their study comprised seven human clinical studies. Various stem cell sources were employed in these studies, including bone marrow-derived MSCs and adult stem cells from sources like the iliac crest. All research successfully achieved bone regeneration utilizing stem cells, but with varying methods and outcomes. It was concluded that tissue-engineering therapy using stem cells to rehabilitate patients with bone atrophies is successful according to the reviewed research [28].

A comprehensive evaluation of the literature was conducted by Fatmah et al. Nine research papers were included in this systematic review, the majority of which described the use of various types of stem cells to effectively treat cleft lip and palate. Mesenchymal stem cells have been shown to be the most effective form of stem cell, followed by adipose stem cells, in the treatment of cleft lip and palate and bone regeneration. However, other types of stem cells have also demonstrated encouraging outcomes [29]. 

## 4. Discussion

This umbrella review aims to offer an overview of the current landscape of stem cell therapy in treating cleft lip and palate (CLP) patients, specifically focusing on bone regeneration. Despite the limited number of studies available, significant findings have been highlighted, particularly regarding the efficiency of stem cells in bone regeneration.

The most effective course of treatment is surgical repair, which offers the best long-term functional and cosmetic results. However, it usually requires several procedures and relies on the surgical team’s expertise and timing. Speech therapy and orthodontic treatment are essential adjuncts that optimize the benefits of surgery. Prosthetic devices may improve quality of life, but they are only meant to be temporary fixes. Psychosocial support deals with the emotional and social difficulties related to CLP and is a crucial part of holistic care [8,9,10]. The anomaly can be successfully treated by the presented non-stem cell treatments for CLP, and stem cell therapy proves to be promising in the future.

Overall, the use of stem cells has shown positive outcomes. However, the most used type of stem cell was mesenchymal stem cells. In all five studies, it was proven that mesenchymal stem cells were the most effective. Martín et al. noted that MSCs, bone marrow-derived MSCs, stem cell culture-conditioned medium (MSC-CM), adult stem cells from the iliac crest, and bone marrow -derived MSCs, along with platelet-rich plasma used for alveolar bone repair, have successfully achieved bone regeneration [28]. Additionally, a study by Sepanta et al. reported that the use of mesenchymal stem cells and growth factors led to considerable bone repair [27]. Likewise, in a notable study by Abbasi et al., important insights into enhancing bone regeneration by the combination of recombinant platelet-derived growth factor and in vitro osteogenic-differentiated human bone marrow mesenchymal stem cells (hBMMSCs) were demonstrated particularly in the repair of alveolar cleft defects. However, most studies included in this research were animal studies, with a limited number of human studies. It was concluded that more clinical follow-ups were essential to demonstrate the credibility of stem cell therapy when used orally [26]. Supporting that, it was stated by Berebichez-Fridman and Montero-Olvera, as well as Nancarrow-Lei et al., that bone marrow-derived MSCs are considered the gold standard of mesenchymal stem cells as they exhibit multipotency and renewal capacity [21,22]. Furthermore, Fatmah et al.’s study reported that several types of stem cells effectively treated cleft lip and palate [29]. It has been proven that mesenchymal stem cells were the most effective, followed by adipose stem cells. Also, the systematic review by Badr et al. noted promising outcomes in the management of cleft patients [25]. However, more advanced clinical trials are required to provide us with reliable information about the application of ADSCs in the treatment of cleft palates. Adipocyte stem cells have been compared with bone marrow-derived mesenchymal stem cells, as reported by Rada, Reis, and Gomes. Even though they may be easily obtained from both sources, bone-derived MSCs are still regarded as the gold-standard osteoprogenitors [23]. On the contrary, George T.-J. Huang stated that embryonic stem cell features have been regarded as the gold standard in pluripotent stem cell-based regenerative therapy [30]. The absence of identical genetics between donor and recipient cells is the most significant disadvantage of human ES cells for therapeutic use. iPS cells overcome this challenge. More critically, many genetic illnesses are uncommon and lack animal research models. Custom iPS cells may be developed from patients and investigated utilizing in vitro or in vivo techniques. As mentioned before, IPSCs do not face controversies like embryonic stem cells. They can differentiate into embryonic stem cells [31]. Although their molecular equivalency is still questioned, it raises the need to further research their use and effectiveness. However, they have the potential to make a valuable change in the field of stem cells. Most studies emphasized the need for further investigation and more clinical follow-ups. Some also mentioned that its effectiveness, predictability, safety testing, and a standardized protocol are required to provide us with reliable evidence concerning stem cell use in cleft lip and palate patients.

### 4.1. Key Findings of This Umbrella Review

1. Mesenchymal stem cells (MSCs) emerged as the most used type of stem cells across the reviewed studies. Martín et al. demonstrated successful bone regeneration using various MSC sources, including bone marrow-derived MSCs and stem cell culture-conditioned medium [28].
2. Sepanta et al. reported significant bone repair outcomes with MSCs and growth factors [27].
3. Abbasi et al. showed promising results in enhancing bone regeneration through the combination of recombinant platelet-derived growth factor and osteogenic-differentiated human bone marrow MSCs [26].
4. Despite animal studies, Abbasi et al. concluded that more clinical follow-ups are necessary to establish the credibility of stem cell therapy in oral applications [26].

The reviewed studies collectively underscore the effectiveness of MSCs in bone regeneration, aligning with the notion that bone marrow-derived MSCs are considered the gold standard due to their multipotency and renewal capacity [21,22].

- While adipose stem cells (ADSCs) also demonstrated efficacy, more advanced clinical trials are warranted to establish their reliability in CLP treatment [25,29].
- Rada, Reis, and Gomes compared ADSCs with bone marrow-derived MSCs, highlighting the latter’s superiority as osteoprogenitors [23].
- The potential of induced pluripotent stem cells (iPSCs) and embryonic stem cells in regenerative therapy was discussed, with considerations for their molecular equivalency and ethical concerns [30,31].

### 4.2. Limitations of the Study

There are two main limitations in this study. The fact that the number of relevant systematic reviews that matched our inclusion criteria was limited could affect our study’s robustness and may limit the breadth and depth of evidence synthesis. Definitive conclusions could not be reached due to variations among treatments and outcomes. Another limitation is the lack of meta-analyses in the included systematic reviews, which could have provided valuable insights into the validity and quality of evidence across various therapies and outcomes.

## 5. Conclusions

This umbrella review presents a comprehensive overview of stem cell therapy in patients with cleft lip and palate (CLP), particularly the use of mesenchymal stem cells (MSCs) and adipocyte stem cells (ADSCs). These specific types of stem cells have promoted bone regeneration and reconstruction in individuals with CLP deformities. The examined studies highlight the potential of stem cell therapy as a complementary approach to existing treatments for CLP. However, there are challenges that need to be addressed, including concerns regarding safety, variations in methodologies, and the need for standardization. Exploring the potential of other stem cell types may further enhance treatment outcomes for CLP patients. Future research should focus on optimizing the use of these alternative stem cell sources, improving the understanding of their therapeutic potential, and overcoming current challenges in clinical applications, particularly in regenerative medicine and bone regeneration.

## Figures and Tables

**Table 1:** Inclusion and exclusion criteria.

No.	Inclusion	Exclusion
1	English language of publication	Languages other than English
2	Studies including stem cells	Studies using other regenerative methods
3	Systematic reviews with or without meta-analysis	Randomized controlled trials, cohort, literature reviews, and case-control studies
4	In vivo studies and human studies	In vitro studies
5	Published between 2014 and 2024	Studies not within the specified range

**Table 2:** Risk of bias (ROBIS) of the included studies.

Review	Phase 2				Phase 3
	1. Study Eligibility Criteria	2. Identification and Selection of Studies	3. Data Collection and Study Appraisal	4. Synthesis of Findings	Risk of Bias in This Review
Badr et al. (2022) [25]	Low risk	Low risk	Low risk	Low risk	Low risk
Abbasi et al. (2017) [26]	Low risk	Low risk	Low risk	Low risk	Low risk
Sepanta et al. (2017) [27]	Low risk	Low risk	Unclear	Unclear	Unclear
Martín et al. (2023) [28]	Low risk	Low risk	Low risk	Low risk	Low risk
Fatmah et al. (2022) [29]	Low risk	Low risk	Low risk	Low risk	Low risk

## Data Availability

All data are present in the article and additional information can be provided when asked.
